# A preliminary assessment of food policy obstacles in California’s produce recovery networks

**DOI:** 10.1007/s10460-022-10407-1

**Published:** 2023-03-17

**Authors:** Cristina Chiarella, Yulia Lamoureaux, Alda A. F. Pires, Rachel Surls, Robert Bennaton, Julia Van Soelen Kim, Suzanne Grady, Thais M. Ramos, Vikram Koundinya, Erin DiCaprio

**Affiliations:** 1 Earth and Life Institute, Université Catholique de Louvain, Louvain-la-Neuve, Belgium; 2 Department of Human Ecology, College of Agricultural and Environmental Sciences, University of California-Davis, Davis, CA 95616 USA; 3 Department of Population Health and Reproduction, School of Veterinary Medicine, University of California-Davis, Davis, CA 95616 USA; 4 Cooperative Extension Los Angeles County, University of California, Los Angeles, CA 91808 USA; 5 Division of Agriculture and Natural Resources, University of California, Los Angeles, CA 91808 USA; 6 Cooperative Extension Bay Area, University of California, Hayward, CA 94541 USA; 7 Division of Agriculture and Natural Resources, University of California, Hayward, CA 94541 USA; 8 Cooperative Extension, University of California, Novato, CA 94945 USA; 9 Division of Agriculture and Natural Resources, University of California, Novato, CA 94945 USA; 10 A Program of Petaluma People Services Center, Petaluma Bounty, Petaluma, CA 94952 USA; 11 Department of Food Science and Technology, College of Agricultural and Environmental Sciences, University of California-Davis, 1 Shields Avenue, Davis, CA 95616 USA

**Keywords:** Food insecurity, Food waste, Food recovery, Gleaning, Regulation

## Abstract

California is a landmark setting for studying produce recovery efforts and policy implications because of its global relevance in agricultural production, its complex network of food recovery organizations, and its environmental and public health regulations. Through a series of focus groups with organizations involved in produce recovery (gleaning organizations) and emergency food operations (food banks, food pantries), this study aimed to deepen our understanding of the current produce recovery system and determine the major challenges and opportunities related to the produce recovery system. Operational and systematic barriers to produce recovery were highlighted by both gleaning and emergency food operations. Operational barriers, such as the lack of appropriate infrastructure and limited logistical support were found to be a challenge across groups and were directly tied to inadequate funding for these organizations. Systematic barriers, such as regulations related to food safety or reducing food loss and waste, were also found to impact both gleaning and emergency food organizations, but differences were observed in how each type of regulation impacted each stakeholder group. To support the expansion of food recovery efforts, participants expressed need for better coordination within and across food recovery networks and more positive and transparent engagement from regulators to increase understanding of the specifics of their unique operational constraints. The focus group participants also provided critiques on how emergency food assistance and food recovery are inscribed within the current food system and for longer term goals of reducing food insecurity and food loss and waste a systematic change will be required.

## Introduction

The rise of supply and demand of rescued food poses enormous logistical challenges in relation to food safety, food loss and waste (FLW) reduction, and associated regulations. Millions of pounds of recovered produce and produce donations from growers (commercial produce farmers), distributors, grocery stores, and farmers’ markets are rescued by gleaners, food pantries and food banks. These produce recovery entities sort, store, and transport vast quantities of produce and directly distribute it to consumers or to other food aid organizations. Adding to the complexity of the produce recovery system, laws that regulate FLW and food safety at the federal, state, local, and organizational level are in place and are continuously evolving. The logistical hurdles related to produce recovery, the potential for regulatory impacts on produce recovery, and the current mechanisms of integration of produce recovery into the food system have yet to be explored on a local level to assess the success or potential for improvement of produce recovery to minimize produce loss and waste and improve food security. This study aims to describe the produce recovery network in California, identify current challenges in the operation of emergency food organizations and food recovery initiatives, and explore the role of policies and regulations in addressing them. Understanding the functional aspects of produce recovery, the various stakeholders involved in the process, and the role of policy and regulation will allow for a more informed evaluation of the current system in reducing produce loss and waste and the contribution to the broader goal of food security and equitable food distribution.

To study these questions, we adopt an transdisciplinary systems approach to research the unique perspectives of different stakeholders in the produce recovery network. Qualitative data was collected from seven in-depth focus groups held with 37 stakeholders working in food banks, food pantries and gleaning groups. Focus group participants included two key stakeholder groups within the emergency food system– (1) gleaners which are informal volunteer groups and nonprofit organizations that harvest, collect, and redistribute fresh produce and (2) food banks in which we included participants from both large-scale food banks with the capacity to distribute large volumes of grocery products across their network of partners, as well as small site-based food pantries within their networks. Focus groups explored the practices and challenges of their operations and how they are affected by different regulations.

In this article, we contend that food banks and food recovery organizations offer invaluable benefits by addressing food security, building community and rescuing produce. However, there are several barriers to their operations, mainly in terms of operational bottlenecks and regulations, that prevent produce recovery programs from optimizing their impacts. Moreover, the emergence of these grass-roots gleaning operations highlight the lack of state support to address both food insecurity and its roots in poverty. Several structural problems within the food system impacting access to fresh produce for the food insecure and FLW are discussed which go beyond the mission of the organizations involved in produce recovery.

### Organization, regulations, and barriers to produce recovery operations in California

#### The role of food recovery in addressing social and environmental impacts of food loss and waste (FLW)

Food recovery efforts in the United States (US) have grown significantly over the last two decades, becoming more foundationally recognized as a core to local food systems. In 2020, four billion pounds of groceries were prevented from becoming food waste and more than 40 million people were served through a network of 200 food banks, showing yearly increases in rescued food supply and demand (Feeding America Annual Report 2020). The rise in food charity has occurred at the same time that FLW has reached unprecedented levels. Food loss occurs in the food supply chain, and includes food leaving the system during production, harvest, distribution, and warehousing (FAO 2019). In contrast, food waste occurs at the retail and consumer level (FAO 2019). Almost a third of the food produced globally intended for human consumption is lost or wasted every year (FAO 2019), and in the US, household and food service food waste reaches 123 kg (271 pounds) per capita, per year, which is higher than other countries of similar economic development (United Nations Environment Programme 2021).

Prior to the World Health Organization (WHO) declaration of the COVID-19 pandemic in March 2020, an estimated 690 million people globally were classified as undernourished, a number that was predicted to increase to around 800 million in 2020 (FAO 2020; WHO 2020; FAO 2021). More recent data indicates that more than two billion people globally experienced food insecurity in 2020 (FAO 2021). Food insecurity has significantly widened among adults in the US during the COVID-19 pandemic, as enrollment in food assistance programs, which provide food to low-income and food insecure populations in the US, increased by 50–75% during the pandemic (Fitzpatrick et al. 2021). Food insecurity was estimated to have affected 17 million Americans in 2020 (Gundersen et al. 2021).

Recently, national and international agencies have prioritized efforts to reduce FLW and reduce food insecurity. In 2015, the United Nations General Assembly (UN) launched the 2030 Agenda for Sustainable Development (Flanagan et al. 2019), which focused attention on the issue of FLW as one avenue towards eradicating hunger by 2030. Among United Nations’ member states, both developed and developing countries adopted a set of 17 Sustainable Development Goals (SDGs). Ending poverty, protecting the planet, and ensuring prosperity for all humans were the central themes used to frame the 17 SDGs (Lipinski 2020). SDG 12 aims to “ensure sustainable consumption and production patterns”. Specifically, the third target under this goal (SDG Target 12.3) calls for halving per capita global FLW by 2030 (Lipinski 2020). Without question achieving this target will require significant improvements in the efficiency of the food system by 2030 (FAO 2019; Galanakis 2020; Lipinski 2020).

Beyond the social impacts of FLW, the environmental impacts of over producing are substantial. In California, landfills are the third largest source of methane, and organic waste in landfills emits 20% of the state’s methane (Cal Recycle 2021). Legislation and other initiatives to reduce organic waste methane emissions are aimed to curtail the progressing climate crisis (Cal Recycle 2021). Highly perishable products, such as meat, poultry, fish, dairy, and produce, are the leading types of foods that end up in landfills. As more states and countries adopt legislation to address climate change through FLW reduction, it creates challenges as well as opportunities to recover more food for human consumption.

#### Produce recovery within the US food assistance and emergency food system

Federal food policy related to food redistribution dates to mid-1930’s when the Emergency Relief Administration (ERA) was established by President Hoover in response to the Great Depression (Fyall and Levine Daniel 2018). The Agricultural Adjustment Act of 1933 (now the Farm Bill) was passed by Congress and surplus agricultural products were purchased by the government and redistributed to people in need (Fyall and Levine Daniel 2018). In 1936, the first school lunch program was launched and the first food stamp program was piloted from 1939 to 1943. The Food Stamp Act of 1964 established a permanent government food stamp program and the Food Stamp Reform Bill of 1977 removed food stamp purchase requirements, mandated national standards of eligibility, and set an appropriation cap for the program (Fyall and Levine Daniel 2018; Levey 1980). This federal entitlement program (for which food stamp purchase requirements were eliminated in 1979) is now known as Supplemental Nutrition Assistance Program (SNAP) and continues to have funding challenges due to political debate (Gritter 2015). In 1972, the Special Supplemental Nutrition Program for Women, Infants, and Children (WIC) was established. In the 2021 fiscal year, the federal government spent $111 on SNAP and other related food assistance programs, with the majority of funding (~ 94%) going directly to households to purchase food (CBPP 2022). Most of the remaining funds went to states, which split the cost of administration of food assistance programs with the federal government (CBPP 2022). The number of SNAP participants grew from 37 million/month pre-pandemic to 43 million in June 2020, with an average daily benefit of $4.16 per person (CBPP 2022).

Concurrently with the governmental supplemental food programs, initiatives to incentivize and/or increase food donations to emergency food programs (for example the Personal Responsibility and Work Opportunity Reconciliation Act of the 1990s) required partnerships with non-profit organizations to redistribute these donated foods to those in need through emergency food programs. In 1977, California passed the first Good Samaritan Food Donation Law and the national Bill Emersion Good Samaritan Act followed in 1996. These laws are meant to encourage food donation by protecting donors from liability except in cases “gross negligence” (Fyall and Levine Daniel 2018). While both SNAP and WIC remain in place today, often these programs alone fail to meet the needs of families. In 2020, the average SNAP beneficiary received approximately $4 per day for food (USDA 2020; Bruckner et al. 2021). When these programs fall short of needs, individuals often turn to emergency food organizations such as food banks, food pantries, or other non-profits (Poppendieck 1999; Bruckner et al. 2021).

Food banks are non-profit emergency food organizations that have a primary role to address food insecurity and nutritional needs of clients (Bazerghi et al. 2016). The first non-profit food bank was established in 1967 (Fyall and Daniel 2018). Food banks can distribute donated, rescued, or purchased food directly to individuals or to local partner organizations (e.g., food pantries, soup kitchens, homeless shelters, emergency food and feeding organizations) (Vitiello et al. 2015; Bazerghi et al. 2016; Bacon and Baker 2017). As of 2015, food banks were estimated to provide supplemental food for more than 46 million people, which is one in seven US residents (Bacon and Baker 2017).

While food banks play a role in the food recovery movement, either by receiving recused food or through directly participating in food recovery as an organization, other organizations have a primary focus on recusing food and redistributing this recused food to those in need. These food recovery organizations (also referred to as gleaning or food rescue organizations) vary significantly in their operational structure so one model-type or definition for these organizations is not possible. Regardless of produce recovery-pathway, gleaning operations build community and can address food access, as well as FLW (Sonmez et al. 2015; Lee et al. 2017). The practice of produce gleaning specifically relies heavily on volunteer and/or low-wage employees (i.e., non-profit staff or farm workers) to recover a variety of fruits and vegetables that are subsequently redistributed to provide healthy food options to food assistance clients (Vitiello et al. 2015; Lee et al. 2017).

Produce gleaning operations are diverse, and can include rescue of fresh produce from commercial fields and orchards, home and community gardens, farmers’ markets, and grocery stores thus impacting multiple points across the FLW spectrum (Vitiello et al. 2015). The reliance on voluntary, non-profit workers versus farm workers vary, depending on the size and structure of the gleaning organization. In California, gleaning programs have rescued and distributed millions of pounds of fresh produce to emergency food organizations across the state (Vitiello et al. 2015). The food collected by produce gleaning operations is distributed through a variety of food assistance organizations such as community food banks, food pantries, member cupboards and directly to individuals experiencing food insecurity who “do the picking” (Vitiello et al. 2015). Food banks may also have internal gleaning programs and many are improving capacity to implement local produce gleaning operations to expand their fruit and vegetable distribution (Vitiello et al. 2015; Lee et al. 2017).

While our study design and analysis distinguish between food banks and gleaning organizations, this distinction may be arbitrary because organizations within these two stakeholder groups differ in significant ways depending on their scale, scope, and context. Some organizations are small-scale, informally structured, volunteer-based, and hold ambitious social or environmental values. Others are large-scale, complex organizations that are formally staffed with trained employees including volunteer coordinators leading well established volunteer programs, they have clearly defined operational structures, and their values are targeted to anti-hunger. In terms of scope, some organizations focus narrowly on harvesting fresh produce, while other organizations span a range of activities including the provision of emergency food, food recovery, food redistribution, and/or community empowerment. And the context in which emergency food activities take place may differ in significant ways from urban to rural communities, to communities with differential ethnic, racial, and socio-economic demographics, to communities with vastly different historical relationships to anti-hunger work, community empowerment, and food justice.

#### Federal and state regulations directly or indirectly impact produce recovery in California

Although regulations play an important role in the overall pathway of food recovery, they may significantly limit the food donation process. The regulatory framework of the California food recovery system is complex and plays a crucial role in defining the context of the food recovery operation. This study took place amidst the rapidly changing regulatory landscape, which contributes to the timeliness of the focus group discussion on regulations. Table 1 provides an overview of federal and state regulations with either defined or perceived impacts on produce gleaning at the time focus groups were conducted in 2019. Broadly these regulations are either related to food safety, food donations, and/or climate change mitigation.

**Table 1:** Overview of federal and state regulations with either defined or perceived impacts on produce gleaning in 2019

Federal Regulations		
42 US Code 1791	Bill Emerson Good Samaritan Food Donation Act (1996)	“Good Samaritan Law” Products donors of food from liability
21 CFR 112	Standards for growing, harvesting, packing, and holding of produce for human consumption (2011)	Food Safety Modernization Act (FSMA) Produce Safety Rule. Sets food safety regulatory standards for produce production and harvesting practices
21 CFR 117	Current good manufacturing practice, hazard analysis, and risk-based preventive controls for human food (2011)	Food Safety Modernization Act (FSMA) Preventive Controls for Human Food Rule. Sets food safety regulatory standards for food manufactures
California State Regulations		
AB 1616	Amendment to Health and Safety Code (2012)	“California Cottage Food Law” Allows for certain low risk food processing in home kitchens for retail sale
AB 1826	Amendment to Public Resources Code (2014)	“Recycling of organic waste” Required mandatory recycling of organic waste for businesses
AB 1990	Amendment to Food and Agricultural Code and Health and Safety Code (2014)	“Community Food Production” Defines a “community food producer”
AB 2561	Amendment to Civil Code (2014)	“Neighborhood Food Act” Allows for “personal agriculture” for home use or donation
AB 234	Amendment to Health and Safety Code (2015)	“Community Food Production Act—Modification” Defines a gleaner may sell or donate whole fruit, vegetables, unrefrigerated eggs provided by a “community food producer”
SB 1383	Amendment to Health and Safety Code and Public Resources Code (2016)	“Short-lived climate pollutants” or “SB1383 Food Waste Recovery” Reduce landfill disposal of organics by 50% (year 2020) and 75% (year 2025); Rescue for human consumption 20% of currently disposed surplus food by 2025
AB 1219	Amendment to Food and Agricultural Code, Health and Safety Code, and Civil Code (2017)	“Good Samaritan Food Law” Specifically defines that protections of the act extend to gleaners
AB 2178	Amendment to Health and Safety Code (2018)	Exempts limited service charitable feeding operations from California Retail Food Code

To encourage the donation of food to emergency food operations, federal and state regulations provide protections to those donating and distributing recovered foods. The Bill Emerson Good Samaritan Food Donation Act shields emergency food organizations and their donors from civil and criminal liability should a negative outcome, such as illness, arise from the consumption of donated or recovered food that was provided in good faith (U.S. Department of Agriculture (USDA) 2021). California also has its own Good Samaritan Food Donation Act, which predates the federal Bill Emerson Good Samaritan Food Donation Act, and an amendment to this regulation in 2017 specifically extended the protections to food donations made by gleaners (California Assembly Bill No. 1219 2017).

Even with the protections of Good Samaritan Acts, until very recently, growers and gardeners in California needed to be an “approved source” in accordance with the California Retail Food Code (CalCode) in order to legally donate their produce. The CalCode, an adaptation of the FDA Food Code, is a food safety regulation intended to mitigate food safety risks in local food systems, most notably restaurants and retail food establishments. Commonly held ways to become an approved source in California included a Certified Producer's Certificate for selling at farmers markets, an Operator Identification Number for pesticide use, Organic certification, or Community Supported Agriculture (CSA) registration. However, many small-scale producers, urban farmers, gardeners, and gleaners did not fit into these categories. Starting in 2012, to help these individuals meet the approved source requirement of CalCode, counties piloted voluntary and no-cost certificate programs intended to enhance food safety and promote small-scale produce sales and donations. These programs were received with varying degrees of success and ultimately adoption of these programs was minimal. Following these pilot programs, AB 1990 (2014) and AB 234 (2015) were passed by the California Legislature regarding Community Food Producers, with similar goals to make it easier for small-scale and urban food producers to meet CalCode requirements.

In 2011, the Food Safety Modernization Act (FSMA) was enacted by Congress to improve the safety of the food supply. The US Food and Drug Administration (FDA) established seven foundational rules to implement FSMA. The Produce Safety Rule (PSR) requires covered produce farms to have a food safety program in place and to document adherence to standards outlined in the PSR. The Preventive Control for Human Food (PCHF) Rule applies to all facilities that manufacture, process, pack, or hold human food (21 CFR 117.1). In general, these are facilities previously required to register with FDA under section 415 of the Food, Drug, and Cosmetic Act. This regulation does not apply to farms or retail food establishments. In general, this regulation applies to most food processors selling food in the US. At the time of this study, it was unclear how these new federal food safety regulations would impact the operations of produce gleaners.

Regulations impacting emergency food operations have shifted from locally-administered, innovative policies aimed to stimulate gleaners to increase food donations to feed the hungry, to state-level regulations, focused primarily on FLW reduction strategies to mitigate climate change. A FLW hierarchy was developed by the United States Environmental Protection Agency (US EPA), with “feeding hungry people” as a high priority method for reducing food waste (EPA 2017; Benson et al. 2018). Nine states currently provide food donation tax incentives for consumers and businesses to help meet food waste reduction goals. Five states including California have implemented organic food waste policies in order to reduce the amount of food waste sent to landfills. In 2014, California AB 1826 “Recycling of organic waste,” required that businesses recycle their organic waste. This bill pushed retail food establishments, restaurants, growers, and food processors to find alternatives for disposing of their food waste, including donation or composting. In September, 2016, California Governor Jerry Brown signed into law Senate Bill 1383 (SB 1383) “Short-lived climate pollutants”, mandating a 75% reduction in organic waste entering landfills by 2025 to reduce methane emissions. SB 1383 further benchmarked that not less than 20% of currently wasted edible food should be recovered for human consumption as an additional 2025 target (Cal Recycle 2021).

### Emergency food organizations and food recovery movements in the context of the food system

Two main narratives are typical in the discussion of the integration of food banks and food recovery organizations in food system. The first narrative focuses primarily on the social impacts of these organizations. The organizations are viewed as an emergency resource for individuals experiencing hunger and food insecurity while also having several other community benefits (Hoisington et al. 2001; Levkoe 2006; Cloke et al. 2017). For example, gleaning organizations have been shown to increase access to fresh produce in communities, provide opportunities for knowledge sharing on nutrition and food preparation, and provide social support within the community (Hoisington et al. 2001). Citizen involvement in gleaning activities can also increase adult learning, reclaim community and public space, develop civic virtues, and increase political advocacy (Levkoe 2006). Food banks have been described as “spaces of care” (Cloke et al. 2017) in addition to revaluing food (Lohnes and Wilson 2018).

The second narrative takes a political economic focus, which frames food banks and food recovery programs in the context of free-market capitalism. Here, emergency food organizations are viewed as a mechanism to reduce government spending on anti-hunger and welfare efforts while benefiting the food industry by enabling donation of food for tax incentives (Poppendieck 1999; Riches 2002; Lambie-Mumford 2013). The charity system upon which food recovery relies has been extensively criticized for reproducing inequities and not addressing the root causes of food insecurity and hunger (Poppendieck 1999; Lambie-Mumford 2013; McIntyre et al. 2016; Riches 2018; Messner et al. 2020). Specifically, for further enriching big corporations through donation-tax write-offs (Vitiello et al. 2015), allowing waste disposal cost savings (Lohnes and Wilson 2018), and not guaranteeing the provision of nutrient-dense foods in sufficient amounts for the current demand (Riches 2011; Bazerghi et al. 2016). Food charity programs are also criticized for binding food security efforts to the industrial food system and diverting attention from redistributive reforms that could address structural causes of food insecurity and diet-related diseases, and could alter the balance of power within the food system (Holt Giménez and Shattuck 2011).

#### Operational and systematic barriers to the success of emergency food organizations and produce recovery programs

Despite the important role of the produce recovery organizations, several challenges in day-to-day operations impede the collection and redistribution of produce. Challenges related to consistent supply of produce, such as inadequate donation or supply, competition with other institutions for produce, and high wholesale price for produce, have previously been described (Bucknum and Bentzel 2019; Bazerghi et al. 2016; McIntyre et al. 2016). Produce is highly perishable and therefore challenges related to transportation efficiency, access to sufficient refrigerated storage space, and funding to implement or sustain the operation are a common obstacle (Bucknum and Bentzel 2019; Wie and Giebler 2013; Forssell and Lankoski 2017; Gokarn and Thyagaraj 2017; Bazerghi et al. 2016; McIntyre et al. 2016).

Regulations are broadly recognized as extensively obstructing the reduction of food waste (Gokarn and Thyagaraj 2017). Consistent with our findings, prior research has established that US food recovery-stakeholders identify regulations as unfavorable to their operations. Specific regulations have been shown to inhibit food recovery, for example by preventing donations of food across county lines (Cooks 2019). But more generally, critiques have posed that current policies and entities involved in managing food surpluses and regulating them, mainly government agencies and private businesses, are focused on ensuring the profitability of the industrial food system, and that any policy related to food recovery must not significantly disrupt business operations (Lohnes 2021).

Even though addressing the series of operational challenges and problematic regulations would most likely lead to a smoother food recovery process, several systemic challenges remain when the goal is to address food security and FLW. This study builds on the existing literature critiquing how emergency food assistance and food recovery is inscribed within the current food system and that short-term adjustments to policy and regulation will not adequately reduce either food insecurity or FLW. The three areas of focus that framed our analysis are: (i) the political economy of food donation and recovery, (ii) the funding of food assistance programs, and (iii) the politics of FLW.

#### The political economy of food donation and recovery

Conceptual works that adopt this line of critique have placed the decommodification of food and labor at the core of the problem. Food commodities and the labor used to produce such commodities are devalued in the current food system (Henderson 2004). The devalued food commodities or unsaleable foods are re-valued through donation for increased capital accumulation by large agro-food actors that benefit from reduced tax burdens, artificial prices, reduction in waste disposal costs, and positive brand impact (Lohnes and Wilson 2018). Food banks have been understood as “re-gifting depots” in that “the original gifts of tax breaks, state agricultural research, trade policy, farm subsidies, and avoided disposal costs are given to large farms, food processing companies, and retail chains; the surplus food is then re-gifted to clients of the food bank” (Lindenbaum 2016). This model further entrenches a secondary food system (Tarasuk and Eakin 2005).

#### The funding of food assistance programs

Prior studies have highlighted that the institutionalization of food banks undermines the state’s obligation to end food poverty and address nutritional health (Riches 2002). To better understand how such institutionalization came into place, it is helpful to review the history of public and private sources of funding for hunger relief in the U.S. (Lohnes and Wilson 2018). Since the 1980s, funding sources of food assistance have drastically changed from primarily public sources to a “private food assistance network” (Daponte and Bade 2006). Several factors have been described that contributed to this shift from public funding of food assistance programs to the privatization of these efforts. First, was the Food Stamp Act of 1977, which eliminated food stamp purchase requirements, but also made enrollment eligibility more stringent and set appropriation ceilings (Levey 1980). In 1979, the first year of implementation, the expenditures of the program were projected to far exceed the funding appropriated. In response to this lack of funding, the anti-hunger community mobilized to provide food assistance (Daponte and Bade 2006). This network of emergency food organizations was further galvanized by the Temporary Emergency Food Assistance Program of the early 1980s (now known as the Emergency Food Assistance Program) (Daponte and Bade 2006). This set the current model in which the USDA purchases commodities which are redistributed via food banks and pantries at the local level (Daponte and Bade 2006). Another set of legislation that significantly impacted the SNAP was the 1996 Welfare Reform Act. This further restricted eligibility to SNAP as well reduced the maximum benefit, which increased reliance on emergency food assistance programs (Edwards 2015).

#### The politics of FLW

FLW reduction initiatives prioritize recovery of food for human consumption, which creates pressure on the charitable food infrastructure to deal with increased donation and redistribution of highly perishable commodities (Lohnes and Wilson 2018). Legislation to reduce greenhouse gas emissions, along with potential lost profits associated with FLW, are now inscribing emergency food organizations within a second-tier food system, supplied with food the industry cannot sell (Buzby and Hyman 2012; Cuellar and Webber 2010; Venkar 2011). Highly perishable foods, such as produce, diverted to emergency food organizations due to FLW initiatives may be of poor quality nearing the end of its shelf-life (Teron and Tarasuk 1999; Wilson 1999). Emergency food organizations must either redistribute or dispose of donated product; simply rejecting donation may jeopardize future donations (Tarasuk and Eakin 2005). In this way, dietary diversity, nutritional quality and food safety responsibility is transferred to the emergency food organization. This approach, in which industry or retailers donate highly perishable products to emergency food operations, also overlooks some important causes of FLW (Parfitt et al. 2010; Mena et al. 2011). Over production by industry (food loss at the production or distribution level) coupled with a lack of consumer acceptance or preference for “imperfect” produce (food waste at the retail or consumer level) means these highly perishable commodities may no longer be suitable for consumption at the time of donation.

## Methodology

This study was conducted by a transdisciplinary team of food systems, urban agriculture, and food safety academics, extension agents, and community practitioners. This study originally aimed to assess food recovery needs related to food safety in California. Prior experience of team members related to unforeseen impacts of state and local regulations on produce gleaning operations led us to explore how newly emerging or evolving regulations would impact produce recovery within California. This team was formed due to ambiguity related to the impact of new federal food safety regulations (Food Safety Modernization Act). However, due to the complex scope of the topic, combined with perceived tensions across stakeholder groups, the research team modified their approach to gather broader qualitative data related to produce gleaning and recovery. The team co-created focus group questions, study design, and research protocols based on our firsthand experience with the topics and local knowledge from their community networks and observed focus groups first hand.

### Focus groups

Seven semi-structured focus groups were conducted throughout 2019. The focus groups were held as part of a project to conduct a qualitative study on the food safety needs of gleaning organizations in California, which received prior approval from the Institutional Review Board (IRB) from the University of California (UC), Davis. The design of this study purposely distinguished between emergency food organizations including food banks and food pantries from gleaner groups to investigate the nature of the specific challenges these two groups face, and their role in alleviating food waste and food insecurity. Thus, the focus groups were held with different stakeholders. The same focus group facilitator(s) led the discussion at each focus group, and multiple members of the research team observed the focus groups. The geographic target areas were the northern and eastern San Francisco Bay Area, the Central Valley, and urban Southern California because of the strong gleaning networks with relationships to UC Cooperative Extension in these locations.

Participant names and organizations of participants were kept confidential beyond the facilitated discussion. Participants were selectively invited based on their similar characteristics from a list generated from existing community networks cultivated by the UC Cooperative Extension personnel and community partners. A list of 60 potential participants from 40 organizations was generated, to whom invitation emails were sent out. From this sample frame, 37 participants (from 28 organizations) attended the focus groups. The breakdown of focus groups by stakeholders was as follows: three gleaner groups, three emergency food organization groups, and one gleaner and emergency food organization combined group. In total 24 participants identified as gleaners and 13 participants were from food banks or food pantries. Participants across the focus groups represented various levels of organizational decision-making from volunteer, to coordinator, to leadership, and therefore had a differential understanding of their organization’s operations, regulatory context, and authority to make changes. The focus groups were recorded and transcribed. A set of semi-structured and open-ended questions related to the gleaning supply network, food safety, relationships with other stakeholders, the role of regulation and possible ways of improvement guided the focus groups sessions. The questions utilized in all focus groups are shown in Table 2. While the conversation was intended to focus on gleaning and food safety, participants shared a wide range of information beyond the scope of the original study which is common in focus groups (Franz 2011). Broader findings beyond the scope of the original intention of the study are included here.

**Table 2:** Open ended questions utilized in all focus groups

Supply network (sources of produce, distribution of produce, etc.)
What types of produce gleaning/food recovery activities is your groups engaged in? Include where gleaned food in coming from and where it is going
Food safety
Has food safety of gleaned produce typically been a concern for your organization? In what ways and why?
Has your organization faced any food safety related challenges when distributing or donating your food?
What types of food safety requirements do you have for donations of gleaned or recovered produce? (food banks and pantries only)
Have you/your group/your organization done any education on best practices in food safety or set up any systems to enhance or ensure food safety? (examples: handwashing, record keeping, worker health and hygiene procedures, harvesting procedures, etc.)
How might you–or your organization–define food safety? What does it encompass?
How do you think the safety of gleaned produce is viewed by different stakeholder groups (gleaner vs. regulator; food bank vs. regulator)? Are there tensions or misunderstanding between the various stakeholders involved in gleaning related to food safety? How do you think the various stakeholders could work together better or differently to support gleaning efforts?
Regulations
Are there any regulatory hurdles or constraints related to gleaning? What food safety regulations have or will have the most impact on produce gleaning operations? Do any recipient organizations have food safety requirements that are a barrier to your operation?
How could regulatory agencies play a more positive role in supporting gleaning?
Current and future needs
What scientific information does the gleaning community need that is not currently available related to the safety of gleaned produce?
Are there specific training or education topics related to food safety that would benefit the gleaning community?

### Data coding and analysis

Focus group recordings and transcripts were first analyzed by two independent researchers, using two sequential First Cycle coding methods: In Vivo coding followed by Structural coding. Researchers decided to use two coding methods in the initial stage to gain a richer perspective of the focus group transcripts through different ways of processing the information. First, In Vivo coding was used to identify themes that drew from the participants’ own language (Saldaña 2013). This method was chosen because of the open nature of the questions asked in the focus groups, which allowed for personal interpretations given the unique circumstances of each participant (their role within the food recovery network, experience, context). Such a first pass at the focus group transcripts allowed researchers to summarize the information from the transcripts into shorter phrases using the participants’ own words, without further interpretation. This process allowed researchers to place the participants’ statements in a more nuanced context (Manning 2017), to later gain a deeper understanding of the broad scope of the information provided. Once the full transcripts were codified through the In Vivo process, structural coding was applied. Structural coding allows for content-based or conceptual classification (Saldaña 2013). This method was chosen as a second First Cycle coding method to make sense of the ideas behind the participants’ statements by grouping the information conceptually, and to move beyond the particular circumstances of participants so that the identified concepts applied to the broader food recovery network. The structural coding process allowed for a classification of the participants’ responses (summarized through the prior In Vivo process) into conceptual classifications, that were grouped into broader topics of main themes and sub-themes (or parent and child codes). The broader themes were grouped such that they responded to the research questions and allowed for further analysis within and across topics (MacQueen et al. 1998), still without attributing meaning or interpretation to the codes. Throughout the two First Cycle coding process, the two independent researchers met regularly to check inter-coder reliability (Saldaña 2013). Although there were some differences in how codes were labeled linguistically, the researchers established an agreement level of 80–90% in their codes which is considered an acceptable level of agreement between independent coders (Saldaña 2013).

Once the two First Cycle coding processes were completed, tables with the resulting themes were shared with the broader interdisciplinary team, who was in charge of making sense of the First Cycle codes through a Second Cycle coding method, Pattern coding. Through the Second Cycle coding process, the team was able to reorganize the information by merging conceptually similar codes, dropping infrequent or marginal codes, and developing a meta synthesis of the First Cycle Codes. The team identified emergent themes, explanations, and attributed meaning to the prior summary of the data using the Pattern coding method (Miles and Huberman 1994), such that fewer themes were determined. Through this process, the team convened on six overarching codes that responded to the research questions of this study. For each of these codes, the team agreed on a description of the code, a summary of the concept behind the code, and exemplary quotes that illustrate the concept described, from both the gleaners and food banks’ focus groups. The resulting and final coding output is in Table 3.

**Table 3:** Thematic codes, descriptions, and exemplary quotes from focus groups with gleaners and food banks

Code	Description	Code summary	Gleaners’ quotes	Food banks’ quotes
Contradictions within the food system	Contradictions and lack of logic within the food system (how gleaning and food banks are inserted within the food system)	Contradictions within the food system and with different food recovery organizations generate a misalignment of goals. Gleaning and food recovery act as an antidote to food waste and hunger and operate with few resources, while the root causes of food waste and hunger are not addressed	“[The County Environmental Health Department] wanted to prioritize nutrition and education, that’s why they said gleaning was not contributing to health. There is a misalignment of goals.”	“We’re helping the hungry and we’re helping reduce food waste. But why not look at why people are hungry and look at why there’s so much food waste.”
Structural and operational challenges	Structural and operational challenges to food recovery	Issues that impede gleaning from reaching its full potential include: institutional obstacles; lack of education and awareness about gleaning; difficulty interacting with many partners; challenges related to scale; lack of a cohesive network and coordination across sectors; lack of sufficient funding	"It's like a delicate dance of like how many policies can be put into place (…) because it's eliminating who is going to be able to receive [food donations]. " (gleaners have a limited number of agencies who are able to receive, more policies might eliminate what agencies are able to receive)	"[Food retailers] could manage their system and give financial assistance to us or give us loads of food rather than us having to deal with almost garbage."
Tensions between sectors	Description of the tensions within and between sectors	Tensions within gleaning and food bank sectors, as well as with other sectors including consumers, producers, industry donors, & volunteers. Examples include gleaners left out of food banks' redistribution efforts because of their small scale; food banks can see each other as competitors; farmers can worry that if they support gleaning efforts, consumers won't pay for their produce	There is a] huge discrepancy between policy maker and person who needs the food, especially for the regulation. There’s one regulation that mandates that agencies, non-profit or church in charge of food distribution need to adhere to qualifications and get permits through the Environmental Health Department. In theory it seems like a good idea, but in practice, it never works out."	"We are seen as a behemoth of an operation, and we are a large organization." "We’re trying to be a good partner but at the same time we have real things to address."
Role of regulations	Perspectives on the impact of regulations, including direct and indirect negative impacts	While some regulations related to food waste have the potential to benefit gleaners' work, many regulations negatively impact gleaning and food banks, including regulations on food safety, gleaning, recipient agencies, zero waste, and composting	Quote 1: "When donors were supposed to register their backyards [with their local County], half of the donors stopped donating. You might get “safer” food, but it's actually not as healthy as it could’ve been." Quote 2: "There is under-utilization of resources for gleaning because of regulations and policies"	"There is the assembly bill about charitable organizations having to get a permit. We’ve always had one. But quite a few of our outreach partners don’t have that in line. That means additional costs, or it may even mean they are not able to continue forward."
Issues with regulators	Challenges with regulators, regulatory agencies, and policy makers	Some regulators don't understand how charitable food distribution works (although a newer generation has a broader perspective of the food system). Regulatory agencies regulate potential problems, not actual ones because there is little understanding of how food recovery operates on the ground. Regulators need to get involved in food recovery efforts to better understand unique context	"If there’s a regulator, they should go do it instead of sitting in an office. In 10 s they will tell you there's nothing wrong with it."	"There is a policy-driven need to standardize food safety practices in these community environments, without understanding how it looks like in on the ground."
Opportunities for improvement	Ideas on how main structural, operational, and regulatory issues could be addressed	Integration of gleaning into the food system would expand capacity, increase effectiveness, facilitate cooperation across sectors, and could improve the regulatory environment. In this way, small-scale and disarticulated food recovery efforts could come together to tackle systemic problems of food waste and food insecurity more comprehensively. For example, by mapping networks, joining forces with other efforts, coordinating logistics, providing better jobs, and proactively improving the regulatory context	"Gleaners have been doing this for decades, they have knowledge of parts of the system (growers, urban farms, chicken, bees, composting systems). There is a need for a collective database and plug them into larger systems"	"Neither we or gleaning are a perfect operation but we do have a common goal. Coming together is important to distribute parts of the scale and tackle the problem comprehensively." "We wrestle with how do we put ourselves out of business. A broader conversation could be good jobs."

### Limitations

While the focus groups drew from the current list of emergency food organizations and gleaners in four metro-regions, this qualitative study is not designed to be representative of the food recovery movement broadly. As with most qualitative studies, this research is designed to understand in-depth processes practiced by specific organizations. Hence, the findings of the study do not have external validity by design. This sample size was purposely selected for in-depth discussions to inform about crucial pathways and challenges in the work of a subset of the emergency food organizations and gleaners within UC Cooperative Extension’s networks (Larson 2004).

## Results

### The produce gleaning supply network in California

Focus group data provided insights on local gleaning supply networks, commonly shared practices and efforts and differences between food bank and gleaner partnerships. Participants shared their community food recovery operations, food sourcing, distribution, types of produce gleaned and other local activities. The primary scope of operational activity for food banks and gleaners includes collecting or receiving recovered food from the source, sorting and transporting it for redistribution, though many additional activities may be undertaken. Food banks are more likely to temporarily store food before distributing it, while gleaners rarely have the capacity or infrastructure to store food.

**Fig. 1:**
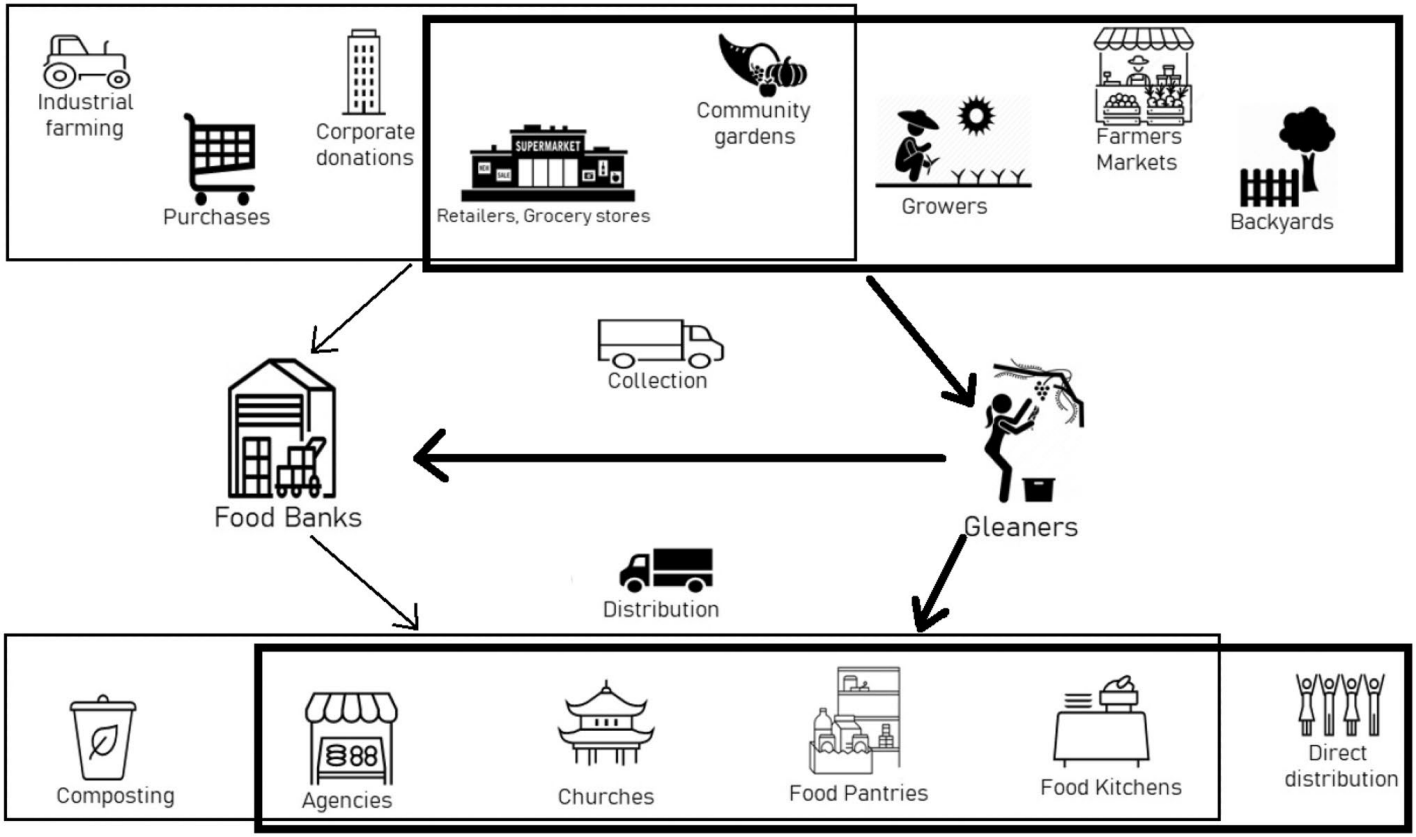
The food recovery supply network for food banks and gleaners. Thin lines and arrows represent food banks’ activities and thick lines and arrows, gleaners’ activities

There are a wide range of ways that fresh produce and other foods move through the emergency food system. Figure 1 illustrates the variety of sources of collection and distribution sites for both emergency food organizations and gleaners based on focus group data collected. Participants from food banks mentioned sources of fresh produce including growers or large packing houses, community gardens, direct or subsidized purchases, farmers markets, corporate donations from retailers or grocery stores, other food banks, USDA commodities, nongovernmental organizations (NGOs), and community food drives.

Gleaner participants, on the other hand, reported collecting produce mainly from sources such as the backyards of small private donors, community gardens, urban farms, farmers markets, wholesale or retail businesses including packing houses, grocery stores and restaurants, research plots or seed companies’—fields, or other donation sites. Many sources identified overlapped between emergency food organizations and gleaner participants. For example, emergency food organization participants report receiving produce from community gardens, although community gardens are not the primary source of their donations. Gleaner participants also report receiving donations from grocery stores and retailers, but not as a main source of their donations, as larger donations from grocery stores and wholesalers are most often directed to food banks. Shared patterns of gleaning activities depend on a variety of factors including the presence of food recovery efforts in the area, relationships between gleaning groups, food banks, food pantries, and other recipient organizations, transportation, and the capacity of these groups to accept gleaned produce.

Food banks distribute mainly to their network of partner agencies which include food pantries, schools, community markets, food kitchens, and faith-based sites. Food banks also divert produce beyond its shelf life to animal feeding operations or for composting. Gleaners distribute mainly to direct service providers including hunger relief agencies, after school programs, senior centers, faith-based sites, food pantries, and in some cases to nonprofit groups making value-added products, such as jam. Unlike food banks the participants from gleaning operations in this study did not report diverting produce to animal feed or to composting. However, due to the nature of relationships with donors, the same pressure to accept lower quality (spoiled) produce exists for gleaners as well. Participants from food banks reported not routinely receiving donations from gleaning organizations because of their own internal requirements, they encourage smaller donations to go directly to local pantries in their network, or because they consolidate smaller donations with larger ones.

Discussions also highlighted some less common gleaning models. For example, some food pantry participants in the focus groups described a model of “farm-to-pantry” in which food pantry volunteers pick up directly from the source; this was particularly true for rural communities where food pantry staff had relationships with local gardeners and growers. Gleaning participants describe several special gleaning models such as “direct pick and keep” in which volunteers harvest and then keep what is harvested. Other special models mentioned included emergency food agencies providing their own volunteers to help harvest, or a special program where growers are funded to pay farm workers to glean their fields for seconds after their main harvest operation.

### Types of produce gleaned in California

The types of produce most often gleaned are hard-fleshed fruits (examples: oranges, apples, lemons, etc.) from backyard trees. Gleaners shared that soft-fleshed fruits (examples: plums, apricots, figs, etc.) that more easily crush, bruise, or squish, are gleaned less often because, in their experience, food banks are hesitant to receive, store, and distribute large volumes of fruit requiring careful handling. Leafy greens were mentioned as produce that is rarely gleaned due to difficulty in their transportation, storage, and shelf life. Some gleaner participants reported rescuing small amounts of non-perishable food from grocery stores or retailers. Participants from both gleaning organizations and food banks identified striving to keep focus on distribution of healthy food and the desire to avoid accepting donations of soda, chips or other processed foods with less nutritional value.

### Additional activities apart from food recovery

Beyond the primary functions of food collection and distribution, food recovery organizations conduct a number of administrative responsibilities to sustain their operations. Food banks, food pantries, and gleaning organizations are responsible for maintaining their sources of funding and food donations. Across all groups (food banks, food pantries, and gleaners), participants reported having to constantly work on attracting new donors, fundraising, grant writing, coordinating access to federal funding, and managing large numbers of volunteers. Despite hurdles with upkeeping complex emergency food supply chains due to inadequate human/vehicle resources, food banks often must seek more diverse revenue streams to maintain the same levels of operational delivery.

Additionally, gleaner participants reported having to develop their own databases or contact lists of harvesting sites and needing to reach out individually to donors prior to harvest. Gleaner participants also mentioned assuming an educational role for showing the importance of growing healthy, safe produce to the donors they work with. Notably, volunteers play a central role in the operations of both the gleaning organizations and emergency food organizations. However, the main challenge identified by participants related to the volunteer workforce for both emergency food organizations and gleaners lies in the difficulty of predicting volunteer availability due to the unpaid nature of their work.

### Structural and operational challenges within produce recovery: Limited financial resources and operational constraints

One of the main challenges identified by both food banks and gleaning organization participants is limited financial resources and operational constraints to address the scale of the problem (Table 3). Participants recurrently stated phrases such as “*A corporate model is required from us, but we have no resources”,* “*It is great that we have been written in the solution (of food waste), but we need the actual resources”,* “*We are playing a huge role, but I don*’*t feel we*’*re getting similar resources put towards our efforts to manage it”*. Food bank participants highlighted the disparity between the limited food waste reduction resources available for their organization compared to the scale of the food industry. Another issue food bank participants raised was the need to compost poor-quality produce that is donated to them and the limited available funding for managing this task. Sufficient financial resources are needed for food recovery operations including transportation, fuel, maintenance, refrigeration, storage, waste management, and staffing and these are challenges for both emergency food and gleaning organization participants.

### Contradictions within the food system

Several participants mentioned that the central logic of food recovery within the conventional US food system needs to be examined, highlighting that the system is fundamentally flawed if so many individuals need food while food is being wasted (Table 3). For example, participants mentioned that their operations are just a band-aid, but that the system needs to produce less food and emergency food organizations need more capacity to address hunger upstream. As one of the participants mentioned, “*We*’*re helping the hungry and we*’*re helping to reduce food waste. But why not look at why people are hungry and why there*’*s so much food waste.”* Participants also expressed the need to come together and think critically about what it takes to end food insecurity and that it is not through produce recovery alone; it is important to consider logistics, integrity of the pipeline, value client voices, and center goals around healthy and culturally relevant food. As a participant stated regarding the goals of food assistance, *“We also wrestle with how we put ourselves out of business.”.*

Another issue raised by both food bank and gleaner participants is the quality, healthfulness, and safety of food donated. Many participants commented that retailers are motivated to donate food only after all options to sell it are exhausted. This sentiment is qualified by a statement from a participant, “*First there*’*s discounted produce, then more discounted produce, then the produce finally ends up at food banks and often it is such poor quality it then is animal food or compost.”* Several participants noted there are no minimum quality criteria for produce donated. As a participant stated, “*The lack of regulations makes you wonder: is this just a capitalist way of moving products?”.*

### Tensions between sectors

There was a lack of consensus with participants in the focus groups regarding existing tensions between sectors in the produce recovery network (Table 3. Some gleaner participants did not experience tensions between regulatory agencies and gleaners. As a participant stated, “*there would be tensions if food was from a* “*waste stream”, but this isn*’*t.”* In contrast, other participants from gleaning organizations reported tensions with regulatory agencies, policy makers, and food industry donors. For example, numerous participants indicated there are tensions with policy makers because of food safety regulations. Gleaner participants indicated that existing local and state regulations related to sources of food that can be gleaned and other food safety requirements introduce unnecessary challenges that inhibit connecting food to those that need it. Participants reported that in practice, these regulations are difficult to comply with and since Good Samaritan Laws protect parties donating food, spending their limited resources on regulatory compliance seems unnecessary. Gleaner participants mentioned that they do not see tensions with consumers or with growers and community gardens. They have dialogues and function as a network of mutual aid. However, they do recognize some ways for improvement, such as building a collective database, better organization, institutionalization within the food system, integration across non-profits and entry into the agro-tourism sector.

Emergency food organization participants reported some tensions with gleaning organizations since food banks and pantries can be perceived as competition or because smaller gleaning organizations may be left out of larger recovery efforts. Participants also identified tensions with the food industry because of the poor quality of the food that is sometimes donated. Because their activities depend so much on their relationship with a few large wholesalers and retailers, they simply cannot reject large quantities of bad produce in fear of jeopardizing the relationship and potentially losing the source of food donation. Participants from food banks reflected on how all stakeholders can address food insecurity better by tackling the problem comprehensively. Participants stated the need to approach this issue collaboratively: to distribute responsibilities and “*make it easier, more streamlined, more integrated into the community, and get more resources”.*

### The role of regulation in produce recovery

Food bank and gleaner participants aligned in the opinion that regulations impact them in significant ways (Table 3). Both types of organizations indicated that their operations are affected by the same types of regulations. These regulations include those aimed to increase food donation (“Good Samaritan Law”), minimize food safety risks (California Retail Food Code (CalCode), Food Safety Modernization Act), or minimize climate pollutants/recover food waste (California Assembly Bill (AB) 1826, California Senate Bill (SB) 1383). Although similar regulations govern their activities, regulations have differential impacts due to their variation in scale and food recovery networks. For example, gleaning organizations are more sensitive to regulations in general due to their small scale and limited operating budget. For gleaner participants, regulations obligating the donors to register with their local enforcement agency (typically county environmental health or agricultural department) were a main concern because they formalized what were typically informal activities. As a participant states, “*When donors were supposed to register their backyards, half of the donors stopped donating. You might get* ‘*safer*’ *food, but it is actually not as healthy as it could*’*ve been.”*

Furthermore, California Assembly Bill (AB) 2178 which exempts limited service charitable feeding operation from uniform health and sanitation standards for retail food facilities^1^ was perceived as irrelevant to gleaner participants due to their reliance on volunteer workforce as the main source of labor and the altruistic nature of produce donations. Gleaner participants were concerned that the various local and state regulations would mandate them to track donations and in some locations in California, local Departments of Environmental Health did enforce traceability requirements either as a precursor to AB 2178 or after the law was enacted. Gleaner participants mentioned that real or perceived differential interpretations and enforcement of regulations have a chilling effect on gleaning activities. When there is ambiguity on whether regulations apply to gleaning organizations, this may reduce participation due to the time requirements for investigating regulation application to the organization, frustrations related to recruiting and maintaining relationships with donors when requirements for donors are unclear, and a general sense of anxiety related to potential regulator interference in the food recovery program.

Some food bank participants expressed concerns with California Senate Bill (SB) 1383 intended to reduce short-lived climate pollutants by reducing the amount of disposed organic waste^2^ Participants were concerned about its possible interference with their food recovery operations. Food bank participants were also concerned about SB 1383 leading to retailers donating more low-quality produce that would normally be sent to a landfill. To minimize penalties, retailers may be pressured to donate produce well past its shelf life. As a participant states, “*They [retailers] are providing more food to us because they don*’*t want to dump it.”* Similarly, a participant shared an experience of large retailers donating significant quantities of spoiled bananas in pursuit of avoiding the penalties of disposing of organic matter into landfills. As a result, the food bank was put in an unfortunate position of having an excess amount of spoiled produce, and not enough funding to dispose of the organic matter. While these specific examples highlight the unintended impact of SB 1383 on emergency food organizations, other participants shared concerns about this regulation leading to donation of low-quality products.

A number of participants in the gleaner group expressed concerns about the lack of knowledge on the part of policy makers about the specifics of on the ground charitable food distribution. Some participants recommended that policy makers “*spend a day observing the work of gleaners”* in order to “*understand how charitable food distribution works”*. This issue was raised several times in both gleaners and food bank focus groups, which points to its significance. Participants also expressed that regulators need to understand that there is “*no one size fits all”* regulation. Both food bank and gleaner participants outlined that funding should increase proportionately to the increased regulations. Gleaner participants also articulated that they should not be held accountable for other people’s growing practices which are perceived as outside of their control. Furthermore, gleaner focus group participants suggested that regulators should be considerate of the volunteer driven and trust-based nature of gleaners' operations and create policies and regulations in accordance with the specific realities of such operations. Collaboration between entities was also referred to as an “*excellent resource”* for the training of policy makers. For example, one participant explained, “*a new generation of regulators that sees food safety in an environmental context rather than just fulfilling the letter of the law”* has been making its way into policy making.

## Discussion

This study captures unique perspectives from those working in both small- and large-scale produce gleaning organizations, food pantries, and food banks. There are important overlaps and distinctions in how participants viewed regulations, obstacles, opportunities for improvement, and collaboration. Perspectives were dependent on the individuals’ organization (food bank, food pantry, or gleaning organization), role within that organization, the organization’s size, and role within the local food recovery system, plus interactions and relationships with local regulators. The categorization of organizations into either ‘gleaning’ or ‘food banks’ factored in our study design and data analysis due to our assumptions on affinity groups and potential desire for confidentiality. After reviewing the data, members of the research team are less certain of the importance of these distinctions. Overall, our findings underscore the structural problems encompassing food donation-sourcing, and funding challenges described previously in varying food systems contexts and frameworks (Forssell and Lankoski 2017; Gokarn and Thyagaraj 2017; Palimaru et al. 2022).

When considering participants’ engagement in the food recovery system, there are significant overlaps in the types of beneficiaries these organizations serve, but significant differences in the sources of produce. Although gleaners and emergency food organizations share the primary goal of alleviating hunger, their operations differ in scale, funding streams, and the nature of their engagement with the emergency food supply chain. Gleaners are mostly small nonprofit organizations with limited government funding and largely depend on volunteer labor. The fluidity of their sources of produce depends on relationships of mutual trust and shared values with donors and recipient agencies. In contrast, food banks are larger food recovery and distribution organizations mainly supported by philanthropy, grants, Feeding America, and federal hunger-relief funds and fellowships. These organizations are highly reliant on food donations, but the nature of some of their donor-relationships is more formal due to the larger scale of food industry donor operations and tax incentives and regulatory fines driving their interests in donating food.

These differences in scale of produce sources have important consequences on the type of constraints and challenges food recovery organizations face in their operations, complications in the produce recovery chain, and how they are differentially affected by regulations. Gleaning organizations are differentially impacted by regulations, such as food safety regulations requiring traceability. Gleaning operations are highly informal, mostly volunteer-based, and rely upon relationships of mutual trust from their produce donors. More stringent food safety regulations may reduce the number of donors in their networks or capacity of their informal and all-volunteer efforts to rescue surplus produce. Gleaning organizations not only address food waste and food insecurity, they are also highly integrated in the community providing interactive education about local, healthy food and opportunities for community empowerment. Therefore, expanding food safety regulation may diminish the critical role these organizations play in their localities. While food banks often receive governmental support, gleaners do not have similar mechanisms to fund their operations and rarely receive financial support or fiscal incentives from the government to help them contribute to food assistance and reduce food waste. This responsibility can add burdens in terms of waste disposal fees, space, labor, volunteer management, and potential ancillary food safety considerations.

Food banks, on the other hand, face different challenges when it comes to regulations. While the food industry can receive tax benefits to support their food waste reduction efforts, similar financial resources are not available for emergency food organizations to reduce food waste that is passed on to them. As food waste is statutorily required to be diverted from landfills due to new greenhouse gas reduction goals required by California Senate Bill 1383, added costs of composting low-quality produce is an important part of food banks’ operations, but fiscal resources to do so at scale are not available. Although food banks appreciate the ability to provide fresh produce, the current system often leads to the donation of lower-quality produce or unhealthy foods. Food banks face two main problems as a result. First, food bank participants fear that by not accepting all kinds of food donations, wholesalers or retailers may not donate again. Hence, they feel forced to accept lower-quality donations, which feeds into the second problem, the lack of resources to dispose of spoiled produce. Food banks currently lack resources for the transportation of large volumes of spoiled produce to animal feeding operations or composting facilities. They also lack sufficient financial resources to pay for increased municipal waste disposal fees.

Our findings on the systemic issues reported by participants directly relate to three structural barriers to the success of food recovery organizations previously identified in the conceptual framework, namely: (i) the political economy of food donation and recovery, (ii) the funding of food assistance programs, and (iii) the politics of FLW. The perspectives of participants in this study underscore the misalignment of goals between food recovery organizations, the food industry, and regulating institutions. The primary goal of food recovery and emergency food organizations is to feed the hungry and in hopes of fulfilling this aim these entities are reliant in many regards to the industry using them as a secondary stream for product they cannot sell directly to their customers for profit. The privatization of welfare has led to organizations with limited financial resources supplementing minimal public efforts to address food insecurity. The current system while functionally serving immediate emergency food needs fails to address the root cause of food insecurity by not providing mechanisms to uplift people from poverty. Moreover, regulations targeted to industry such as those focused on food safety or FLW are impeding progress toward to goal of minimizing food insecurity held by food recovery and emergency food organizations. The politics of FLW have inadvertently, or perhaps not, transferred significant responsibility to food recovery and emergency food organizations to ‘be part of the solution’ while leaving them underfunded and diverting attention from the root causes of FLW which include overproduction and the organization of the industrial food complex, among others.

## Conclusions

This study finds several important obstacles in enhancing accessibility of high quality, nutritious, and safe produce through food recovery. An important common obstacle identified by participants is long term funding. Emergency food organizations are responsible for extensive food rescue, transport, distribution, waste removal/composting activities on behalf of large food businesses. This effectively externalizes costs for food businesses and transfers such responsibilities to emergency food organizations/sector, creating an organizational burden. One important opportunity to enhance emergency food system-functionality is increased funding for food recovery and emergency food organizations. This could be through expanded governmental programs and should include direct food industry funding.

We also found important challenges and tensions around the role of regulations for both participating emergency food- and gleaner- stakeholders. Recent food safety regulations can limit organizational capacity to optimize food recovery and redistribution. If such regulatory pressures continue or increase, then under-resourced and informal gleaning organizations may be forced to cease operating. For food banks, problematic regulations are those that compel wholesalers and retailers to donate low-quality food, by limiting the amount of food waste these businesses are able to send to landfills by imposing fines. These regulations risk transferring the problem of food waste to food banks which have few resources to manage the scale of need. Therefore, another opportunity to enhance produce recovery programs is that regulations be fit with the scale, practices and operational capacities of organization(s) or groups recovering gleaned produce and externalities not be passed on to the charitable feeding sector. Further, we recommend exploring the feasibility and potential benefit of regulations setting standards to ensure the quality, freshness, and healthfulness of donated food. As food waste reduction policies mandate that a proportion of that food be recovered for human consumption (such as CA SB 1383), food recovery networks need to be resourced to meet the scale of this challenge.

Participants across stakeholder groups agreed that to improve the food recovery system, more coordination is needed with all entities along the emergency food supply chain. The engagement of policy makers and regulators with those in food recovery networks would benefit both parties as policy makers and regulators would gain insight into how emergency food systems work, including their multifaceted forms, scales, and practices. Some focus group participants spoke of a potential paradigm shift in policy making in California to support food recovery networks, but more research is needed to identify the changes in policy-making patterns. Moreover, a medium-term opportunity based on this work is a concerted effort between policy makers, regulators, and those within the food recovery system to have open dialogue and resource sharing to promote the success of food recovery efforts.

Our findings also provided a critique on the way in which food recovery is inscribed within the food system and on whether the current structure contributes to minimizing food waste and promotes food security. Specifically, the privatization of food assistance through a network that functions as a dependency of the industrial food system (and their profitability) creates serious issues for social protection, as it essentially hands food security, dietary diversity, and nutrition to small organizations with limited resources. In this process, food industry waste also becomes a negative externality passed on to the charitable food sector. Many participants questioned whether the current system and reliance on charitable donations and volunteer work adequately contributes to addressing FLW and access to healthful foods for all. Some stakeholder participants also expressed concerns that the current food recovery system serves to reinforce a corporately driven food system perpetually incentivizing over-production and over-consumption. All of this points to the need for systemic reform at top levels of the conventional food system, to address equitable food access. Because of the many layers of complexity of the issues discussed, adopting a food system perspective for such reform could help articulate current sparse efforts that tackle different dimensions individually. To properly address the root causes of food insecurity and FLW, a focus on dignified employment and earnings, and on regulating incentives for overproduction are necessary. Such ambitious reform would certainly also require restructuring the sources of funding and redirecting public resources to these needs.

## Footnotes

1 For a full review of California Legislative Information – Assembly Bill No 2178 (Chapter 489). https://leginfo.legislature.ca.gov/faces/billTextClient.xhtml?bill_id=201720180AB2178.

2 For a full review of the Organic waste methane emissions reductions regulation, see CalRecycle, Laws and Regulations, Short-Lived Climate Pollutants (SLCP). https://www.calrecycle.ca.gov/laws/rulemaking/slcp
